# Multi-store collaborative delivery optimization based on Top-K order-split

**DOI:** 10.1371/journal.pone.0278690

**Published:** 2023-03-09

**Authors:** Yanju Zhang, Liping Ou, Jiaxu Liu

**Affiliations:** 1 School of Business Administration, Liaoning Technical University, Huludao, Liaoning, China; 2 Software College, Liaoning Technical University, Huludao, Liaoning, China; Nottingham Trent University School of Science and Technology, UNITED KINGDOM

## Abstract

Regarding the fulfillment optimization of online retail orders, many researchers focus more on warehouse optimization and distribution center optimization. However, under the background of new retailing, traditional retailers carry out online services, forming an order fulfillment model with physical stores as front warehouses. Studies that focus on physical stores and consider both order splitting and store delivery are rare, which cannot meet the order optimization needs of traditional retailers. To this end, this study proposes a new problem called the “Multi-Store Collaborative Delivery Optimization (MCDO)”, in which not only make the order-split plans for stores but also design the order-delivery routes for them, such that the order fulfillment cost is minimized. To solve the problem, a Top-K breadth-first search and a local search are integrated to construct a hybrid heuristic algorithm, named “Top-K Recommendation & Improved Local Search (TKILS)”. This study optimizes the search efficiency of the breadth-first search by controlling the number of sub-orders and improving the initial solution of the local search using a greedy cost function. Then achieve the joint optimization of order-split and order-delivery by improving the local optimization operators. Finally, extensive experiments on synthetic and real datasets validate the effectiveness and applicability of the algorithm this study proposed.

## 1 Introduction

New retailing is one of the trends in traditional retail enterprises’ transformation, which is characterized by the integration of online channels with offline channels. Regarding the online retail order fulfillment optimization, many researchers have focused more on warehouse optimization and distribution center optimization, ignoring the order fulfillment model with physical stores as front warehouses. Online retail orders are characterized by small demand for each item, but a wide variety of items demand and a strict time window for delivery, making it less efficient to use large warehouses or distribution centers to deliver orders in a unified manner. Using physical stores to deliver orders is more in line with customers’ actual needs and helps retailers reduce the cost of deploying warehouse centers. However, the stock of items in physical stores differs depending on the location and storage space of the store, as well as the long-term needs of customers. At the same time, the uncertainty of offline customers’ consumption also leads to real-time changes in-store inventory. Thus, as the size of orders grows, the probability that the nearest store cannot meet the demand for the nearest online orders becomes greater. For this, specific methods are needed to allocate orders to convenient stores and design delivery routes to meet the customer’s delivery time window requirements and the enterprise’s goal of minimizing order fulfillment cost.

### 1.1 Literature review

There are three main aspects about order fulfillment optimization: order allocation optimization, order delivery optimization and the combination optimization of order allocation -delivery. For order allocation, Xu et al. improved order fulfillment through the utilization of delayed demand fulfillment along with retailer-directed demand allocation [1]. Mahar et al. developed a "quasi-dynamic" order allocation strategy that allocates orders to warehouses based on expected inventory, shipping cost [2]. Jasin et al. introduced demand forecasting into the cost function and proposed a linear programming-based multi-commodity order execution scheme [3]. Torabi improved the efficiency of solution search by using a benders decomposition-based approach [4]. To optimize the inventory allocation to different warehouses, Acimovic et al. proposed a heuristic algorithm that considered the overstocking of goods [5]. Zhu et al. further optimized the allocation of goods in online retail warehouses by a heuristic clustering algorithm [6]. With the goal of profit maximization, Lei et al. proposed an inventory allocation strategy that can reduce order-split for online retailers [7].

Order delivery can be further subdivided into order-split delivery and order consolidation delivery. In order to improve the efficiency of order fulfillment, Catalán proposed the idea of splitting deliver orders when out-of-stock items or ordered items are stored in different warehouses [8]. Based on split delivery, Archetti proposed the rule of splitting orders by item type other than by quantity, which improved the efficiency of vehicle delivery [9]. Hanbazazah et al. established a mixed integer programming model to deal with the order-split delivery problem and proposed a three-stage exact solution algorithm to solve the model [10]. Similarly, Pankratz proposed an improved heuristic algorithm to improve the delivery efficiency of orders [11]. Li et al. designed a hybrid genetic algorithm to solve the order delivery optimization problem [12]. Abdulkader et al. used inventory matching to study the secondary delivery between distribution centers and retail stores and solved it using heuristic algorithms [13]. Wang et al. studied the multi-center common delivery problem and designed a hybrid algorithm incorporating ant colony algorithm for solving it [14].

In the study of order consolidation delivery, Stenius et al. considered a single-warehouse, multi-retailer inventory system and proposed shipment consolidation of orders from different retailers through the order’s delivery time [15]. Lai et al. explored the trade-off between expedited orders and transportation costs under an order consolidation strategy [16]. Zhang et al. proposed a method to consolidate sub-orders between warehouses to reduce trans-shipment volumes and transportation costs [17]. Van showed that the implementation of lateral transhipment generated significant cost savings [18]. Meanwhile, Ramakrishna studied an item-warehouse lateral transit model and proposed a search method incorporating greedy heuristics to achieve cost optimization [19]. Bebitoglu et al. also proposed a quadratic mixed integer planning model based on multi-center collaborative distribution [20]. By studying different ways of consolidating inventory and vehicles, Hall found that order consolidation can lead to lower transportation costs, but also increase inventory costs [21].

Since order-split is unavoidable and order consolidation will lead to higher inventory costs, researchers have started to jointly optimize order-split and order delivery to improve the practical application of order-split. Zhang et al. proposed a joint optimization of order-split and logistics delivery for online supermarket, and proposed a two-stage heuristic algorithm to solve the model to reduce the problem-solution space [22]. However, his study did not consider the delivery time window requirement for orders. Rieck studied the problem of order pickup and delivery in the case of multiple items in transit under a two-level distribution system, but the study focused on the warehouse and transit station without involving the store level [23]. Ishfaq improved the efficiency of solving joint optimization problems by making combinatorial decisions for the allocation and delivery of online orders [24]. Ardjmanda et al. constructed an integer programming model for order consolidation and order allocation, then used a multi-objective genetic algorithm to reduce shipping costs and time [25].

The above literature on order fulfillment is almost exclusively focused on warehouse and distribution station studies. While Gao et al. studied the order fulfillment model of buy online and pick up in store (BOPS) and found that BOPS is beneficial for retailers to broaden their customer base [26]. With the advancement of the BOPS model, it is important and relevant to conduct research on order fulfillment for stores. Some scholars have started to conduct order fulfillment optimization research with physical stores as the object. In the problem of order-split and order-delivery in stores, orders have the characteristics of strict delivery time windows, high uncertainty of order demand, low order volume but high demand for varieties of items, etc., which are pretty different from warehouse distribution. Therefore, Zhao constructed a solution model to optimize collaborative drug delivery in the context of new retail based on the space-time network model and changed the delivery subject from a warehouse to a physical pharmacy. Then, used Lagrangian relaxation to reduce the difficulty of solving the problem of collaborative delivery [27].

Furthermore, in the context of online pharmacies with “multi-warehouse in one metropolitan area” and “multi-item order”, Yu proposed an order fulfillment model that used physical pharmacies as front warehouses to deliver items. Then, used an improved particle swarm optimization and a Clarke-Wright saving algorithm to solve the model [28]. Zhang et al. proposed a Tabu search algorithm based on forest search, in order to optimize order allocation and delivery of multiple warehouses, which effectively improved the search efficiency and order fulfillment efficiency of the scheme [29]. Song constructed an order fulfillment strategy selection model with the goal of reducing order fulfillment costs in the context of multi-warehouse cooperative distribution research, and solved the model using linear programming and greedy algorithms [30].

In summary, the research of order fulfillment focuses on warehouses and distribution centers, with less collaboration between different stations in the delivery process. Studies that use the store as the subject of order fulfillment usually use certain stores as transit stations for orders to achieve the goal of uniform delivery of orders, which is liable to increase the workload of the transit stores. Moreover, these studies do not consider the reality that retailers are more likely to adopt a store-to-customer direct delivery model of order fulfillment.

### 1.2 Contribution of this paper

To bridge this research gap, this paper jointly optimizes order-split and order-delivery based on the commonality of research in order fulfillment problems. This study presents an order fulfillment problem, called the “Multi-Store Collaborative Delivery Optimization (MCDO)” problem, in which the physical stores are the delivery agents. Physical stores are the main object of this study. This study not only assign the orders to the appropriate stores, but also design a delivery plan for each store. Through the collaboration among stores, the delivery of multi-commodity orders and achieve the goal of minimum order fulfillment cost can be completed. The main contributions of this paper are as follows.

- This study formulates a problem named the “Multi-Store Collaborative Delivery Optimization (MCDO)” problem, which makes plans and design routes for stores in an online scenario, such that the order fulfillment cost is minimized.
- This study proposes a hybrid heuristic algorithm called “Top-K Recommendation & Improved Local Search (TKILS)” to solve the MCDO problem. Firstly, use a Top-K recommendation to obtain the initial order-split plan, and then an improved local search is used to adjust the initial order-split plans and delivery routes.
- This study conducts extensive experiments on both synthetic and real datasets. Evaluations verify the effectiveness and applicability of the hybrid heuristic algorithm.

## 2 Materials and methods

### 2.1 Problem statement

The “Multi-Store Collaborative Delivery Optimization (MCDO)” problem can be described as follows: Consider there is a retailer with several physical stores in a region. Each store has order fulfillment ability and can collaborate to deliver orders. Each multi-item order can be split into sub-orders based on the storage of each store, as well as the distance between the store and the order. For each item in the order, it can be delivered directly from the store to the customer or transferred to another store for consolidation and deliver to customers with the other items. Use the following example to explain the MCDO problem.

**Fig 1:**
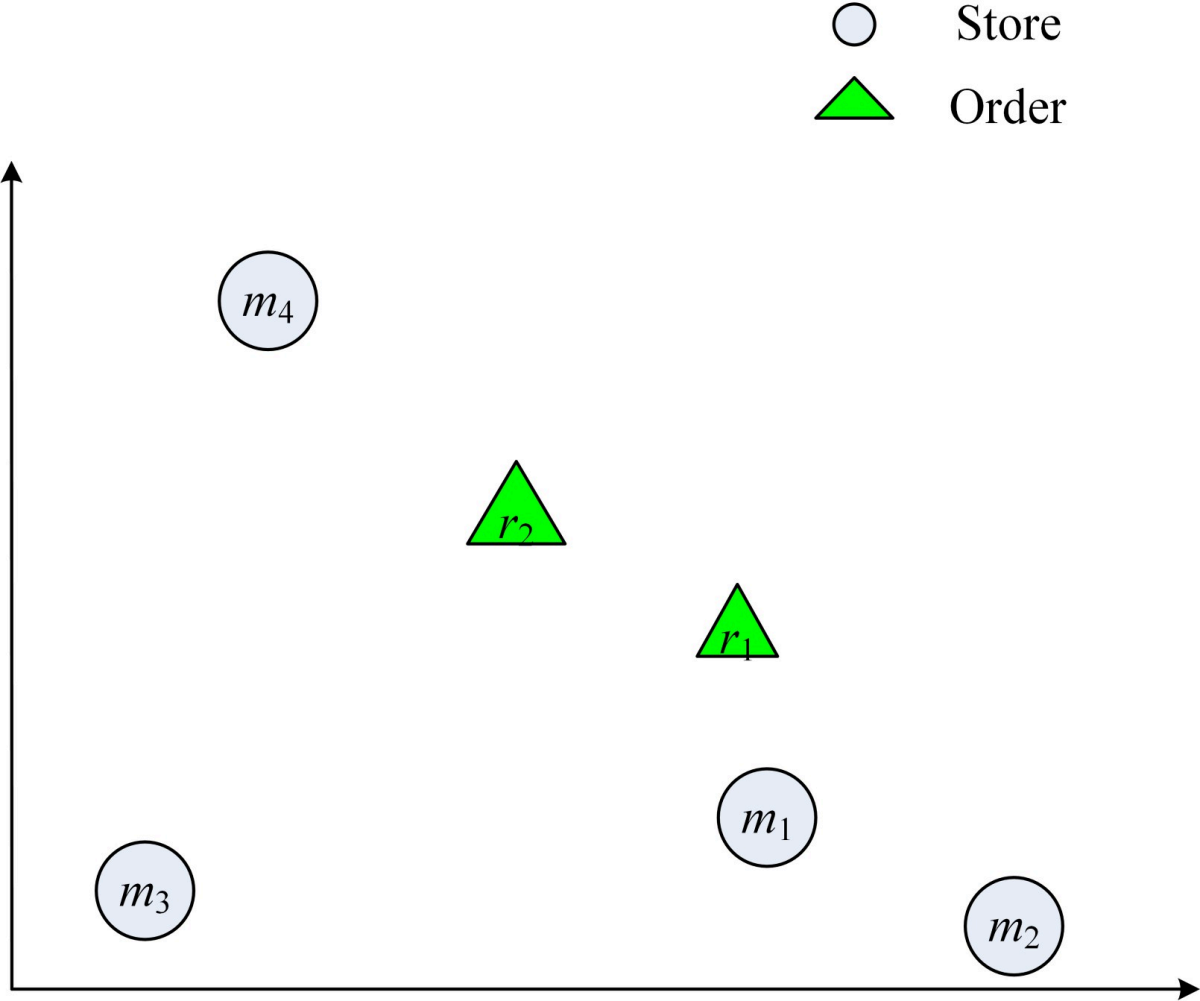
The location of each store and order. Fig 1 is the example diagram of the distribution of stores and orders in the example.

Example 1. Tables 1 and 2 show the information of the order and store, including the arrival time, the delivery time window, and the demand item of the order, and Table 3 shows the distance between the order and the store. Fig 1 shows the location of each store and order, the straight-line distance on the graph represents the delivery time and delivery cost of an order. Denote store and order by *m* and *r* respectively, and use *a*^*m*^ and *a*^*r*^ to denote the items that the store owned and the order required, now there are four stores *m*_1_, *m*_2_, *m*_3_, *m*_4_, and two dynamically arriving online orders *r*_1_, *r*_2_. It is important to match real-time orders with stores, i.e., select stores that can complete the orders at a minimum cost, and design the order delivery routes for stores.

**Table 1:** Order information.

Order	Arrive Time	Time Window	Items required
*r*_1_	0	[7,8]	$a_{1}^{r_{1}}$、$a_{2}^{r_{1}}$、$a_{4}^{r_{1}}$
*r*_2_	2	[8,9]	$a_{3}^{r_{2}}$、$a_{4}^{r_{2}}$、$a_{5}^{r_{2}}$

Describes the example orders *r*_1_, *r*_2_ appearance times, delivery time window requirements, and the types of items demanded.

**Table 2:** Store information.

Store	*m*_1_	*m*_2_	*m*_3_	*m*_4_
Items owned	$a_{1}^{m_{1}}$、$a_{2}^{m_{1}}$、$a_{3}^{m_{1}}$	$a_{3}^{m_{2}}$、$a_{4}^{m_{2}}$	$a_{1}^{m_{3}}$、$a_{2}^{m_{3}}$、$a_{4}^{m_{3}}$	$a_{1}^{m_{4}}$、$a_{3}^{m_{4}}$、$a_{5}^{m_{4}}$

Describes the types of goods stocked in the example stores *m*_1_, *m*_2_, *m*_3_ and *m*_4_.

**Table 3:** The distance between order and store.

Distance	*m*_1_	*m*_2_	*m*_3_	*m*_4_	*r*_1_	*r*_2_
*m*_1_	-	2	4	8	1	3
*m*_2_	2	-	8	10	3	5
*m*_3_	4	8	-	5	5	4
*m*_4_	8	10	5	-	3	2
*r*_1_	1	3	5	3	-	1
*r*_2_	3	5	4	2	1	-

Specifies the delivery distance between each store and the order. The delivery distances in the examples are all derived from this.

**Fig 2:**
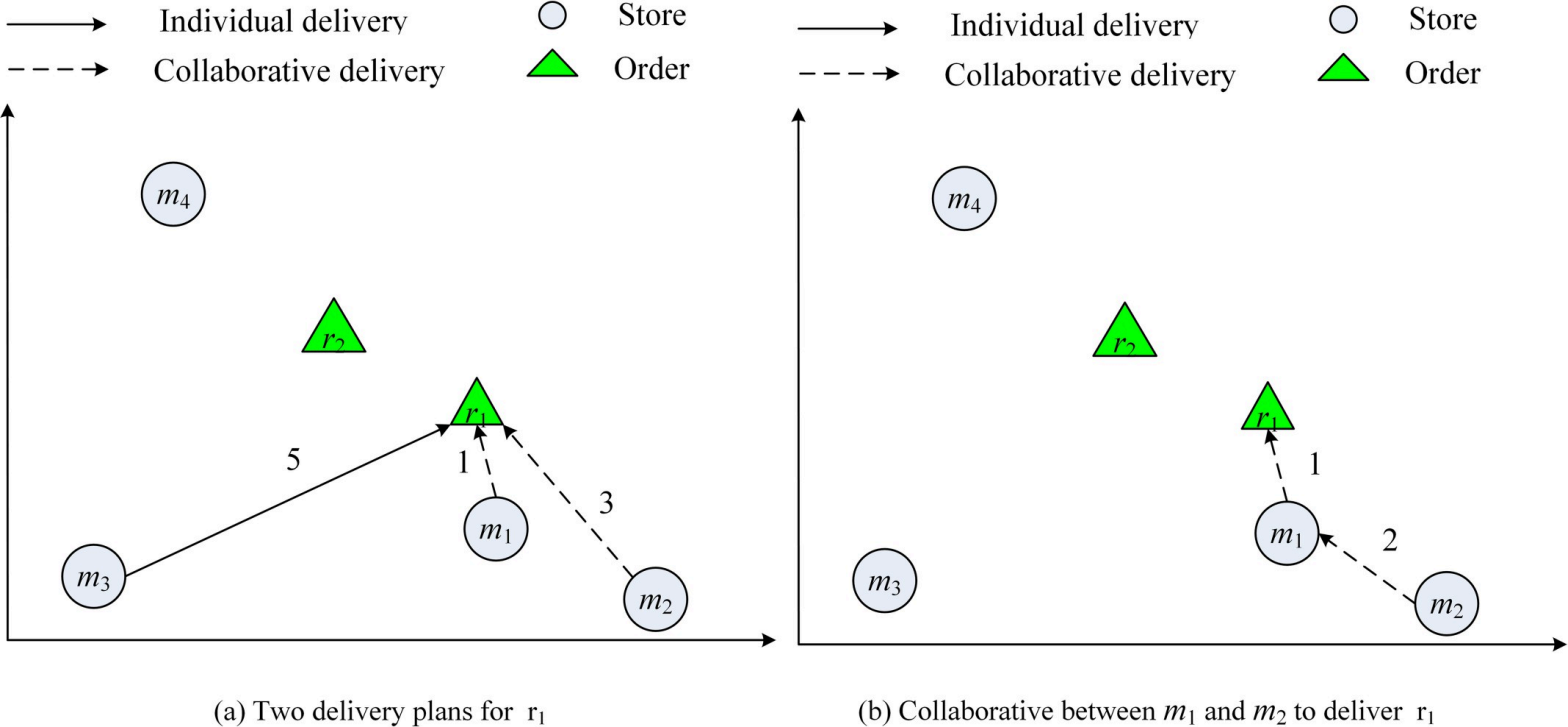
The example of individual and collaborative delivery of *r*_1_. The dotted line represents multi-store collaborative delivery, and the solid line shows single-store delivery.

At time 0, order *r*_1_ arrives, it can be delivered by {*m*_1_, *m*_2_} and {*m*_3_} for two solutions, as shown in Fig 2. The cost of solutions is 4 and 5, and the delivery time of solutions is 3 and 5 respectively. Both solutions satisfy the time window of *r*_1_, thus the optimal split plan of *r*_1_ is, therefore {*m*_1_, *m*_2_}. And with the consolidation of *m*_1_ and *m*_2_, the joint delivery route *S*_*v*_ = {*m*_2_, *m*_1_, *r*_1_}, where the cost and the delivery time are both 3, which is better than the individual delivery, it’s the optimal delivery solution of *r*_1_ in the offline scenario.

**Fig 3:**
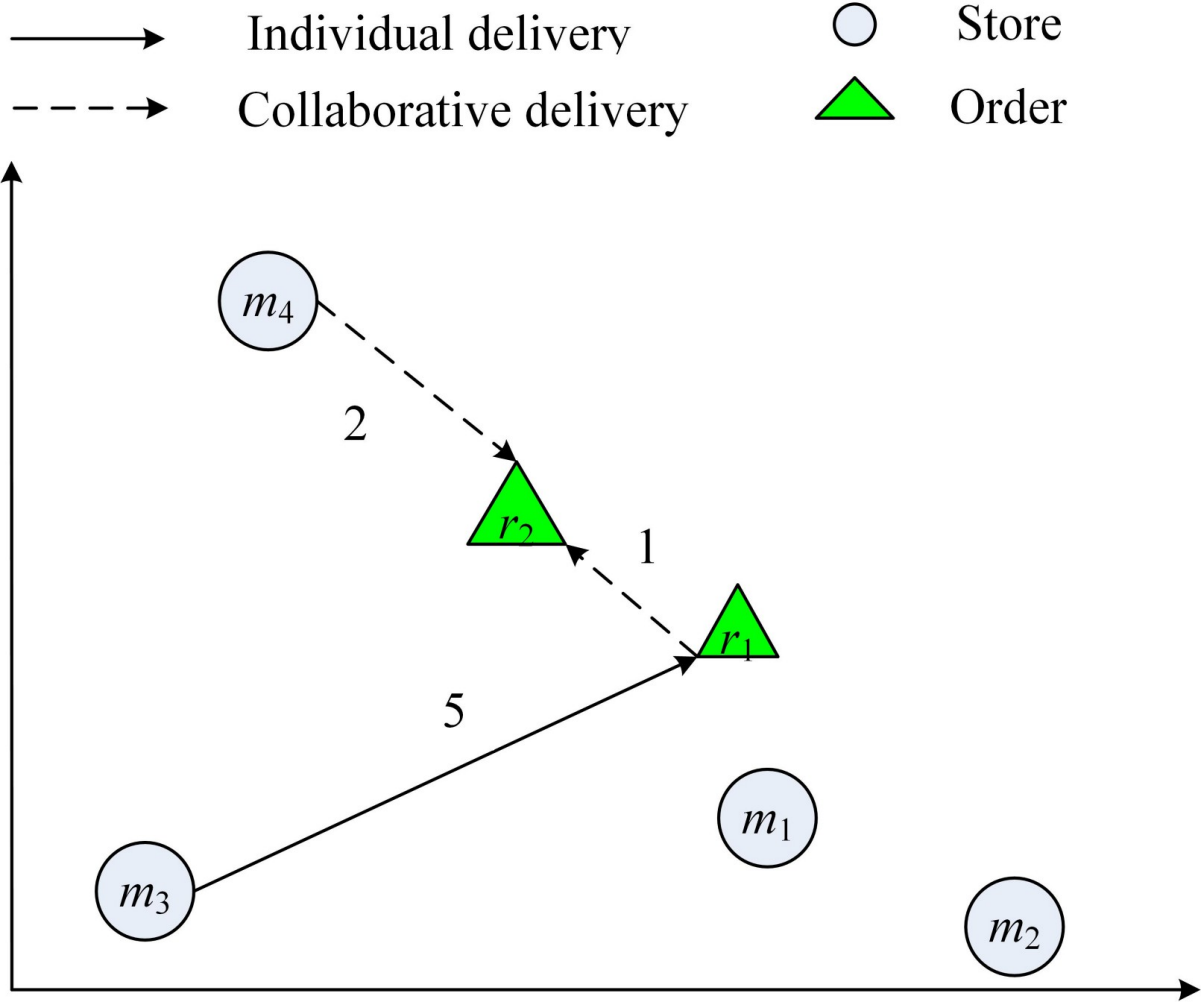
The final delivery plan of *r*_1_ and *r*_2_. The illustration shows the order fulfillment model combining single store delivery and multi-store delivery.

At the moment 2 *r*_2_ arrives, the optimal order-split plan of *r*_2_ is {*m*_3_, *m*_4_}. In this situation, if *r*_1_doesn’t deliver, with the consolidation of stores, the solution of *r*_1_ can be change from {*m*_1_, *m*_2_} to {*m*_3_}. In this way, the total cost of delivering {*r*_1_, *r*_2_} reduced from 9 to 8, and the delivery time of *r*_1_ is 7, *r*_2_ is 8, which both satisfy the time window, the final delivery plan of *r*_1_ and *r*_2_ can be seen in Fig 3. It can be imagined that as the scale of orders expands, the route repetition rate between different solutions will rise. Through the joint optimization of order-split and order-delivery, the route repetition rate can be effectively reduced, which is conducive to improving the efficiency of order fulfillment and reducing the order fulfillment cost of the enterprise.

### 2.2 Model

This study proposes an integer programming model to solve the MCDO problem, in which each item is a commodity and the vertex nodes stand for the stores and the order nodes. To determine the split plans for items in an order by distance between orders and stores, and the commodity stocks, is to find the optimal delivery routes for these items. And to achieve a reduction in order fulfillment costs, the objective function can be constructed as follows:

$$\min C=\alpha \sum_{r\in R}\sum_{v\in V}x_{v}^{r}+\beta \sum_{v\in V}\sum_{i\in B}\sum_{j\in B,j\neq i}d_{ij}x_{ij}^{v}+\gamma \sum_{v\in V}\sum_{r\in R}\left\{\max[(t_{r}^{v}-l_{r}),0]\right\}$$ (1)

Eq (1) aims to minimize order fulfillment costs, it comprises three components:

- $\alpha \sum_{r\in R}\sum_{v\in V}x_{v}^{r}$ is the total fixed dispatch costs of vehicles, among which, $x_{ij}^{v}=\{0,1\}$ denote whether vehicle *v* deliver order *r*, *r*∈*R* and *v*∈*V* mean that the order and the vehicle all belong to the store. *α* represent fixed cost per vehicle created;
- $\beta \sum_{v\in V}\sum_{i\in B}\sum_{j\in B,j\neq i}d_{ij}x_{ij}^{v}$ is the variable cost of vehicles, *d*_*ij*_ is the distance between node *i* to node *j*, if vehicle *v* travels from node *i* to node *j*, then $x_{ij}^{v}=1$, otherwise, $x_{ij}^{v}=0$, *i*≠*j*. In which, *i* and *j* belong to set *B*, set *B* contains all the orders *R* and the stores *M*. *β* is the unit travel costs of vehicle *v*;
- $\gamma \sum_{v\in V}\sum_{r\in R}\left\{\max[(t_{r}^{v}-l_{r}),0]\right\}$ is the penalty cost for delivery overruns, $t_{r}^{v}$ denotes the time for vehicle *v* to complete order *r*, *l*_*r*_ is the latest time to deliver order *r*. For order *r*, if $t_{r}^{v}-l_{r}>0$, then a punish *γ* is incurred.

To ensure that the objective function is valid and realistic, the following constraints need to be made.


$$\sum_{i\in B}x_{ji}^{v}=\sum_{i\in B}x_{ij}^{v},\;\forall j\in B,j\neq i,v\in V$$ (2)

Eq (2) ensures that the inflow and outflow of the vehicles satisfy the supply and the demand requirements at all the nodes in the combined network;

$$\sum_{i\in B}x_{ji}^{v}\leq x_{v},\;\forall j\in B,j\neq i,v\in V$$ (3)

Eq (3) indicates that a node can only be visited at most one time in a delivery route, i.e., $x_{ij}^{v}\leq 1$;

$$\sum_{r\in R_{i}^{v}}\sum_{a\in A_{r}}y_{ira}^{v}W_{r}^{a}\leq W_{v},\;\forall i\in B,v\in V$$ (4)

Eq (4) represents the weight limit of vehicle *v*, if vehicle *v* deliver item *a* for order *r* at node *i*, then $y_{ira}^{v}=1$, $W_{r}^{a}$ denotes the requirement of item *a* by order *r*. At all times, the amount of items carried on vehicle *v* must be less than the maximum capacity of the vehicle, i.e., *W*_*v*_;

$$\sum_{r\in R}y_{ra}^{m}W_{r}^{a}\leq W_{m}^{a},\;\forall m\in M,a\in A_{m}$$ (5)

Eq (5) represent the stock limit of store *m*, if item *a* of order *r* is assigned to store *m*, then $y_{ra}^{m}=1$, $W_{m}^{a}$ denotes the inventory of item *a* in store *m*. At all times, the number of item *a* accepted by store *m* for an order must be less than the store’s inventory.


$$x_{ij}^{v}+x_{ji}^{v}\leq 1,\forall i,j\in B,j\neq i,v\in V$$ (6)

Eq (6) shows sub-loop removal restrictions, it is possible to avoid multiple delivery routes.

### 2.3 Methods

#### 2.3.1 Algorithm design

Jasin has proved that the order-split and allocation problem is an NP-hard problem [12], and the delivery route problem is also a typical NP-hard problem, so the MCDO problem is an NP-hard problem as well. While the exact algorithm takes too much time to solve the problem and is usually only applicable to small-scale problems, the heuristic algorithm can find a satisfactory solution in a short time. And is suitable for large-scale order fulfillment situations, which also has broader applicability.

To this end, a hybrid heuristic (TKILS) algorithm is designed to solve the MCDO problem. The algorithm consists of order-split, order delivery, and joint optimization. In the order-split part, use an improved breadth-first search to reduce the solution space of the problem. In the order-delivery part, use a greedy cost function to generate the initial delivery route. And finally, optimize order-split and order-delivery simultaneously with improved local search.

The specific processes of TKILS are as follows: Firstly, based on the store’s inventory and orders information, the minimum number of split orders and the shortest distance are combined to obtain the Top-K order-split plans. Each order’s Top-1 order-split plan determines the initial order allocation plan. Secondly, according to the initial order allocation plan, the greedy cost function generates the initial order-delivery route. An improved local search algorithm is then used to explore the new neighbourhood space for the remaining Top-K order-split plans. All solutions are stocked in the alternative pool (POOL) to obtain more feasible neighbour solutions. Finally, if no new orders arrive, the satisfactory solution is output directly. If there is a new order, then the Top-K operation is performed on the new order and matched with the solutions in POOL to obtain a new satisfactory solution.

#### 2.3.2 Order-split based on improved breadth-first search

In order-split, the distance between the stores and orders and the number of split orders are the main influences of order fulfillment cost, so this study introduces the idea of breadth-first search. It first sorts stores in descending order according to the order coverage rate $\frac{A_{r}^{m}}{A_{r}}$, and taking the store with the highest coverage rate as the first store to deliver the order. *A*_*r*_ denotes the remaining items demand of order *r*, $A_{r}^{m}$ denotes the item remaining demand for order *r* that can be satisfied by store *m*. In case the order is not fulfilled, continue to search for the next store with the highest coverage to get a new order fulfillment solution and repeat the above steps until get the set of stores that fulfil the order.

**Fig 4:**
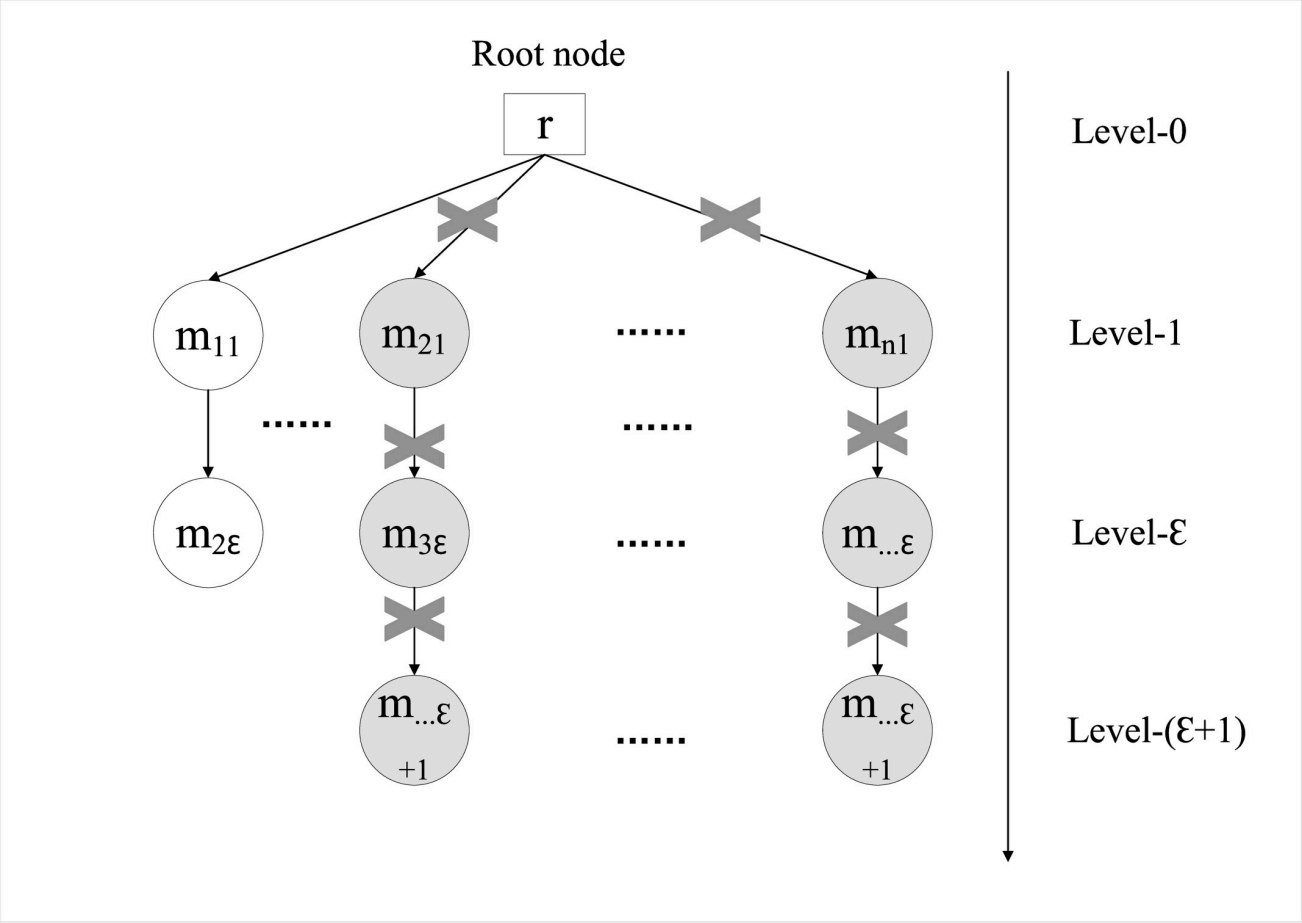
The breadth-first search tree. Note: search the node tree from node *r* of level-0, and when a branch tree does not meet the requirements, delete the branch and continue to search other branch trees.

In order to improve the speed and quality of breadth-first search, a split-order level limit *ε* is presented, since too many split times tend to increase the complexity of the solution. Once the delivery combination search of an order exceeds level *ε*, the node tree is deleted. As shown in Fig 4, use the split of order *r* as an example, each level is sorted according to the order coverage rate $\frac{A_{r}^{m}}{A_{r}}$ from left to right in descending order for each store. The first node tree is searched in order from store *m*_1_at level-1, so named the sub-node *m*_11_, and the order can be satisfied at level *ε*, i.e., sub-node *m*_2*ε*_. The node trees searched from store *m*_2_ and store *m*_*n*_ cannot satisfy the order demand within the level limit *ε*, so stop the search and cut-off the whole node trees.

Use *L*(*z*) to denote the level of node *z*, $\xi_{me}^{r}$ to denote the sub-order of order *r* assigned to store *m* at level-*e*. In a node tree, *L*(*e*)<*L*(*f*) denotes that the search level-*e* is smaller than the search level-*f*, i.e., search stores at level-*e* first, then search level-*f*. $A_{r}^{e}$ shows the remaining demand for order *r* before the search at level-*e*. Then $\xi_{me}^{r}$, the items assigned to each store at each level can be expressed as follow,

If *L*(*e*)<*L*(*f*), then,

$$\xi_{me}^{r}=\{A_{r}^{e}\cap A_{r}^{me}\}$$ (7)

$A_{r}^{e}\cap A_{r}^{me}$ means that at level-*e*, the item assigned to store *m* must be an item owned by store *m* and required by the order *r*.


$$\xi_{mf}^{r}=\{\{A_{r}-(A_{r}^{e}\cap A_{r}^{me})\}\cap A_{r}^{mf}\}$$ (8)

$A_{r}-(A_{r}^{e}\cap A_{r}^{me})$ denotes the remaining demand for order *r* after the search at level-*e*, same as Eq (7), $A_{r}-(A_{r}^{e}\cap A_{r}^{me})\}\cap A_{r}^{mf}$ means that at level-*f*, the item assigned to store *m* must be an item owned by store *m* and required by the order *r*.

If *L*(*e*)>*L*(*f*), then,

$$\xi_{mf}^{r}=\{A_{r}^{f}\cap A_{r}^{mf}\}$$ (9)

$$\xi_{me}^{r}=\{\{A_{r}-(A_{r}^{f}\cap A_{r}^{mf})\}\cap A_{r}^{me}\}$$ (10)

**Fig 5:**
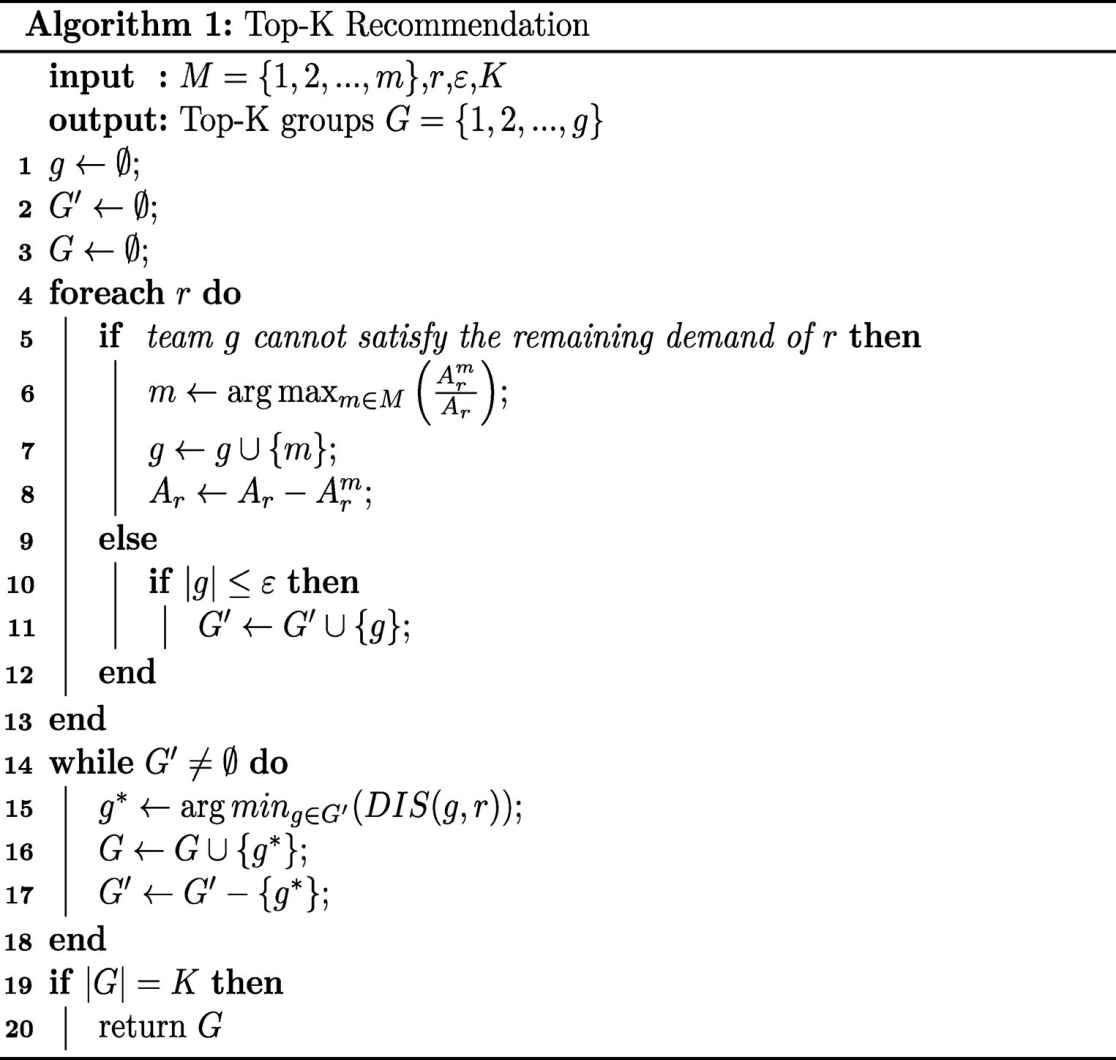
Top-K recommendation. It shows the pseudo code for Algorithm 1.

Based on the idea of breadth-first search, the Top-K recommendation algorithm can be divided into two parts, Fig 5 illustrates the procedure of the Top-K recommendation. The first part (lines 1–13) generates the initial optimal-split plan based on the minimum number of splitting orders. The second part (lines 14–20) obtains the Top-K order-split plans by sorting the plans in ascending order according to the minimum delivery distance.

In lines 1–3, first initialize the order-split plan *g*, the set *G*′ of plans that meet the limit on the number of split orders, and the set *G* of Top-K order-split plans. Then, in lines 4–8, calculate the coverage rate of each store for the remaining demand of the order (*A*_*r*_), the store with the highest coverage rate ($\frac{A_{r}^{m}}{A_{r}}$) is selected as the next store to fulfill the order. Repeat the above steps until the order is completely fulfilled. In lines 9–12, determine whether the order-split plans meet the constraint based on the number of split orders limit *ε*, delete the plan if it does not meet *ε*, otherwise continues to the next step.

In lines 14–20, the plans that meet the constraint *ε* are stocked in the set *G*′ and, if *G*′ is not empty, the plans are sorted in ascending order according to their delivery distance, where the plan with the shortest distance is transferred from the set *G*′ to the Top-K set *G*. Several iterations are performed until the set *G* reaches K order-split plans.

#### 2.3.3 Order-delivery route based on greedy cost function

After obtaining the initial order allocation plan for each store using Algorithm 1, it is then necessary to design the initial delivery route for each store. Considering the delivery cost generated by order insertion and the impact on the delivery time of other orders, a greedy cost function can be used to generate the initial order-delivery route for each store. Then express the greedy cost function in Eqs (11) and (12).


$$\{\begin{array}{l} c(p,r^{1},q)=\eta c_{1}(p,r^{1},q)+\varphi c_{2}(p,r^{1},q),\;\eta +\varphi =1\\ c(m,r^{1})=\eta c_{1}(m,r^{1})+\varphi c_{2}(m,r^{1}),\;\eta +\varphi =1\\ h(r^{1})=c(p,r^{1},q)-c(m,r^{1})\\ \Delta c_{r}=\min[c(p,r^{1},q),c(m,r^{1})]+\cdots +\min[c(p,r^{n},q),c(m,r^{n})],n\geq 1 \end{array}$$ (11)

$$\{\begin{array}{l} c_{1}(p,r^{1},q)=d_{pr^{1}}+d_{r^{1}q}-d_{pq}\\ c_{2}(p,r^{1},q)=t_{q}-l_{q} \end{array}$$ (12)

*c*(*p*,*r*^1^,*q*) denotes the cost incurred where inserting sub-order *r*^1^ of order *r* between location (*p*,*q*) of an existing route *S*_*v*_, *c*(*m*, *r*^1^} means using an idle vehicle *v*′ starts from store *m* to deliver only the order *r*. *h*(*r*^1^) denotes the cost difference between 1) inserting sub-order *r*^1^ into an existing route *S*_*v*_ and 2) using an idle vehicle *v* for delivery. If *h*(*r*^1^)<0, then choose 1) inserting *r*^1^ into *S*_*v*_, otherwise choose 2) using an idle vehicle to deliver *r*^1^. The insertion cost of order *r* equals to the sum of the insertion costs of the stores responsible for delivering the sub-orders of order *r*, denoted by Δ*c*_*r*_. min[*c*(*p*,*r*^1^,*q*),*c*(*m*,*r*^1^)] means to choose a lower cost delivery solution.

*c*_1_(*p*,*r*^1^,*q*) denotes the increased delivery distance after inserting sub-order *r*^1^; *c*_2_(*p*,*r*^1^,*q*) denotes the value of the timeout impact on the delivery time of other orders caused by inserting sub-order *r*^1^. $d_{pr^{1}}$ denotes the delivery distance added after inserting sub-order *r*^1^ after location *p*, $d_{r^{1}q}$ denotes the delivery distance from *r*^1^ to location *p*, *d*_*pq*_ shows the original delivery distance from location *p* to location *q*. *l*_*q*_ denotes the latest arrive (delivery) time at location *q*, and *t*_*q*_ is the time to arrive location *q* after inserting sub-order *r*^1^. If *t*_*q*_>*l*_*q*_, the insertion cost rises. *η*、 *φ* are weighting parameters, both *η* and *φ* can be set to 0.5 since delivery time and delivery cost matter equally. When the constraints (order delivery time window and vehicle capacity limit) are met, insert the order into the best delivery location, and the initial order-delivery routes are formed after several iterations.

#### 2.3.4 Joint optimization of order-split and order-delivery based on improved local search

The original local search uses a randomly generated initial solution to explore the neighbourhood and generate a better solution. Considering that the quality of initial solution has an impact on the number of iterations of the local search, and that initial solutions with poor quality require more iterations to find a satisfactory solution. Use the initial order-delivery routes generated by the greedy cost function as the initial solution to improve the local search. After the initial solution *S* is obtained, delete some stores’ orders firstly, and then generate a series of neighbour solutions S* of the initial solution *S* by adjusting the order-split plans and the order-delivery routes.

All the solutions are stored in the alternative pool (POOL) in ascending cost. Since the orders in the new retail context require real-time response, to improve the efficiency and quality of the insertion of new orders, set the POOL to store up to 300 feasible solutions. If the POOL is full of feasible solutions, it can be updated only when the cost of the new solution is lower than the existing solution according to the principle of minimum cost. The strategy for local search includes order deletion and neighbour solution optimization, as follows.

***1) Order deletion***. For the initial route solution *S* generated by the greedy cost function, the deletion operator randomly deletes 20% of the orders to obtain an incomplete solution *S*′. If the order is delivered by a single store, the order is deleted directly. If the order is delivered by more than one store, then after randomly deleting a sub-order of the order, the sub-orders of the order from the other stores need to be deleted along with it. Set *u* consists of the remaining Top-K order-split plans for the deleted order *r*. A plan is randomly selected from set *u* to complete the delivery of *r* by using roulette. The lower the distance cost, the higher the probability of selecting the plan. For each store in the plan, the new neighbour solution S* is obtained by calculating and inserting *r* into the best position of the optimal route according to the greedy cost function. Finally, the initial solution *S* and neighbour solutions are stocked in POOL.

***2) Neighbour solution optimization*.** To achieve the goal of multi-store collaborative delivery proposed in this paper, the routes of neighbour solutions are optimized by using the exchange operator, the relocate operator, and the 2-opt operator. Before the 0-exchange and 0-relocate, the distance function condition judgment is added. If the distance from store *m* to the cooperative store *m*′ is less than the distance from *m* to order *r*, and the constraints are satisfied. Then the 0-exchange operator is used to generate a new delivery route, at which the vehicle *v* of *m* can go to *m*′ to hand over the sub-order of order *r*. If the constraints are not satisfied and no order arrives, the first solution in POOL and its order-split plans are output directly. If a new order arrives, the Top-K operation is executed on the new order to generate K order-split plans and match them with the solutions in POOL. Then, use the greedy cost function to select the optimal order-split plan and output the optimal order-delivery route.

**Fig 6:**
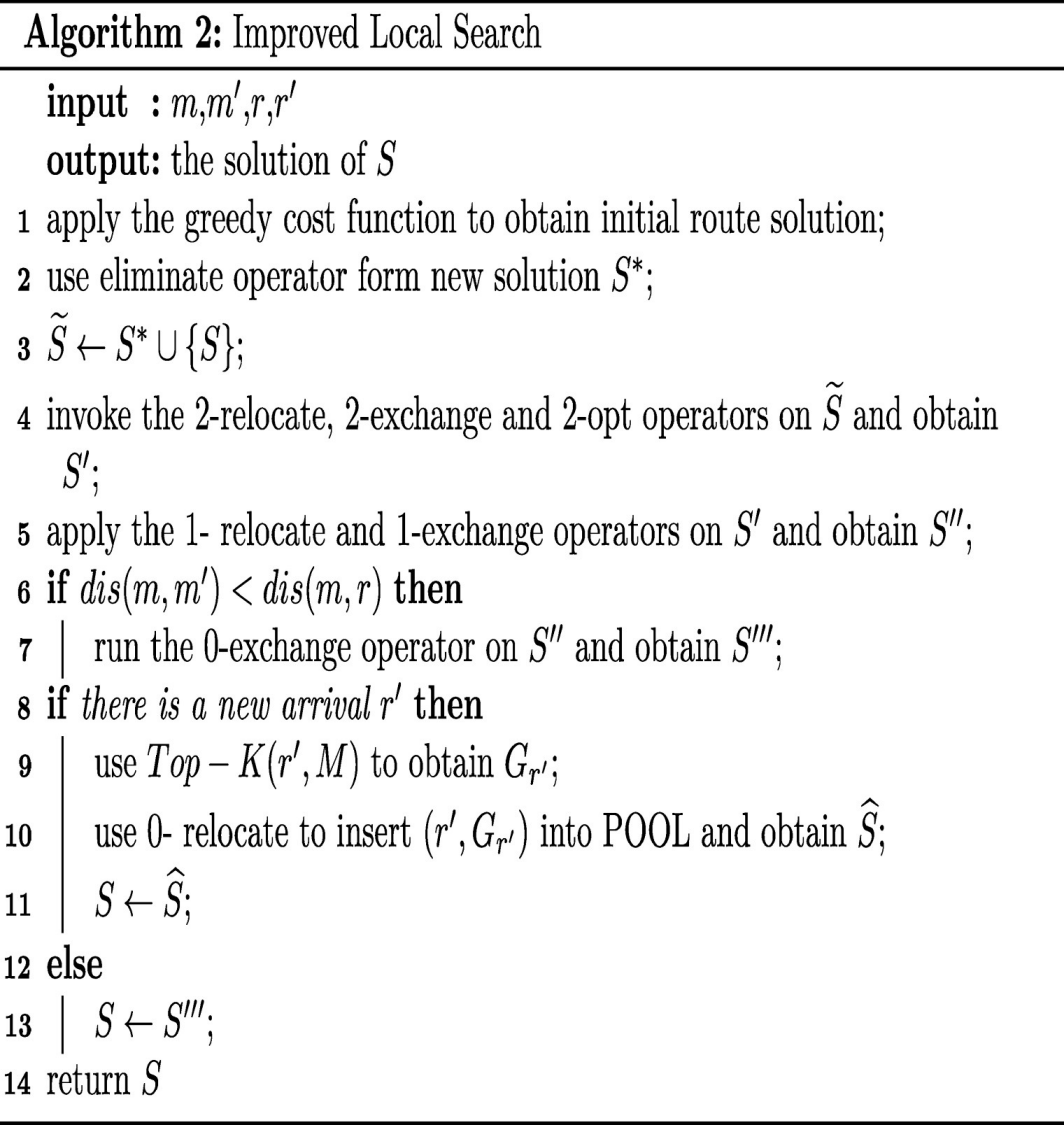
Improved local search. Pseudocode is used to show the running process of Algorithm 2.

Combining the above steps, there is Algorithm 2 Improved Local Search, and the specific steps of Algorithm 2 in Fig 6 are as follows: In lines 1–3, after using the greedy cost function to generate the initial order-delivery route *S* for the initial order-split plan. Use the elimination operator to delete 20% of the orders and construct a series of neighbour solutions S* to obtain the set $\tilde{S}$ consisting of *S* and S*. In lines 4–5, taking store *m* as an example, 2-relocate, 2-exchange, and 2-opt are applied to change orders and delivery queue on the two routes of *m*, to obtain the new solution *S*′. Then by performing 1-relocate and 1-exchange operators on *S*′ obtain the new solution *S*″. Finally, using 0-relocate and 0-exchange operators. In lines 6–7, if *m* and *m*′ deliver order *r* together, and the distance between *m* and *m*′ is less than the distance from *m* to *r*, then apply the 0-exchange operator to generate the latest solution *S*‴.

The above operation is performed for all stores in each run, and many satisfactory neighbour solutions can be obtained by jointing optimization, and the resulting solutions are all stocked in POOL, which facilitates the real-time response of subsequent orders. In lines 8–11, if a new order *r*′ arrives, the Top-K operation is executed on *r*′ to get Top-K order-split plans and match them with the solutions in POOL. The optimal order-split plan is selected using the greedy cost function and inserted into the feasible solution with 0-relocate. The optimal route is finally generated and returns the satisfactory solution *S*. In lines 12–14, when no new orders arrive, *S*‴ is the final satisfactory solution.

The TKILS hybrid heuristic algorithm is obtained according to sections 2.3.1, 2.3.2, and 2.3.3. Using TKILS, the MCDO problem proposed can be handled to solve the online order fulfillment problem. The discuss of the strengths and weaknesses of TKILS is in the next section.

#### 2.3.5 Time complexity

The amortized analysis can be used to analyze the complexity of TKILS. Assume *m* and *n* are the number of stores and orders, respectively. First, the time complexity of Algorithm 1 is *O*(*mn*). In Algorithm 2, the time complexity for calling greedy cost function is *O*(*n*log*n*), the time complexity of lines 2–5 and line 6–7 is *O*(*n*). Due to a call to Algorithm 1, the time complexity of lines 8–11 is *O*(*mn*), thus the total complexity of lines 6–11 is *O*(*mn*^2^). Combing these two parts, the time complexity of TKILS is *O*(*mn*+*mn*^2^).

## 3. Results and discussion

### 3.1 Experimental study

This study will evaluate the performance of TKILS on both synthetic and real datasets. And compare TKILS with K-Nearest Neighbor & Local Search (KNNLS), Breadth-first & Local Search (BLS), Top-K Recommendation & Local Search (TKLS), Top-1 Recommendation & Improved Local Search (T1ILS). Xin proposed the KNNLS, which first assigned orders to the nearest store and then used local search to plan the order-delivery route. It improved the order fulfillment rate, but leads to multiple order splits and higher delivery costs [31]. Han’s BLS aims to reduce the number of order splits by completing order with the fewest stores, it tends to lead to large differences in the quantity of orders assigned to each store [32]. TKLS and T1ILS are variants of TKILS. TKLS uses an improved breadth-first search to generate K optimal order-split plans and then use local search to deliver the orders. T1ILS first generates a best order-split plan and then uses the improved local search to obtain the order-delivery routes.

#### 3.1.1 Synthetic datasets

Table 4 shows the parameter settings for synthetic datasets. The default settings are marked in bold. Specifically, order set (denoted by |*R*|) and store set (denoted by |*M*|) are randomly sampled on a [30km,30km] metric space, and vary the number of |*R*|and the number of |*M*|. This study also changes the number of items in order (|*q*|), and the type of items on order (|*A*|). To compare the five algorithms, the search level limit *ε* and the number of solutions of the Top-K recommendation were both set to 3, i.e., each order can be split into up to three sub-orders, and each order has three lowest cost solution combinations. The arrival time of orders is generated by the Poisson distribution, with a parameter λ = 10/min for orders.

**Table 4:** The parameter settings for synthetic data.

Parameter	Setting
|*R*|	10,50,**100**,200,300
|*A*|	5,**10**, 15,20,30
|*q*|	1,**2**,3,4,5
|*M*|	5,**10**, 15,20,30
*α*	6y
*β*	1y/km
*γ*	24y/h
*ε*	3
K	3

#### 3.1.2 Real datasets

Table 5 shows the parameter settings for the real datasets, which are collected from a retail enterprise located in Shanghai, China. The dataset includes 1,092,103 online order records of customers in Shanghai, China in January, 2021. The records are chosen in a [20km,20km] region during the peak-hour period 18:00–19:00. In this region there are 15 stores. Finally, the real datasets include 1000 orders. Test the orders arrived in each day and choose array [200,400,600,800,1000] as example for comparison of the quality of algorithms.

**Table 5:** The parameter settings for real data.

Parameter	Setting
|*R*|	200,400,**600**,800,1000
|*A*|	4,**6**,8,10,12
|*q*|	1,**2**,3,4,5
|*M*|	3,6,**9**,12,15
*α*	6y
*β*	1y/km
*γ*	24y/h
*ε*	3
K	3

#### 3.1.3 Metrics

Note that all the compared algorithms are differ in the effectiveness and efficiency of order fulfillment due to their differently order-split methods. Hence, this study mainly compares their performances in terms of the running time, running memory and the execution cost under the same experimental context. Here the running time refers to the total time an algorithm takes from receiving an order to the completion of the assignment, running memory is the computer memory occupied by the execution of the algorithm, which affects the algorithm’s computing speed, and the execution cost is the order fulfillment cost of executing the solution obtained by the algorithm.

#### 3.1.4 Implementation

All the algorithms are implemented in MATLAB, and the experiments were performed on a machine with 4 Intel (R) Core (TM) i5-5200H CPU and 512GB memory.

### 3.2 Experimental results analysis

#### 3.2.1 Results on the synthetic datasets

**Fig 7:**
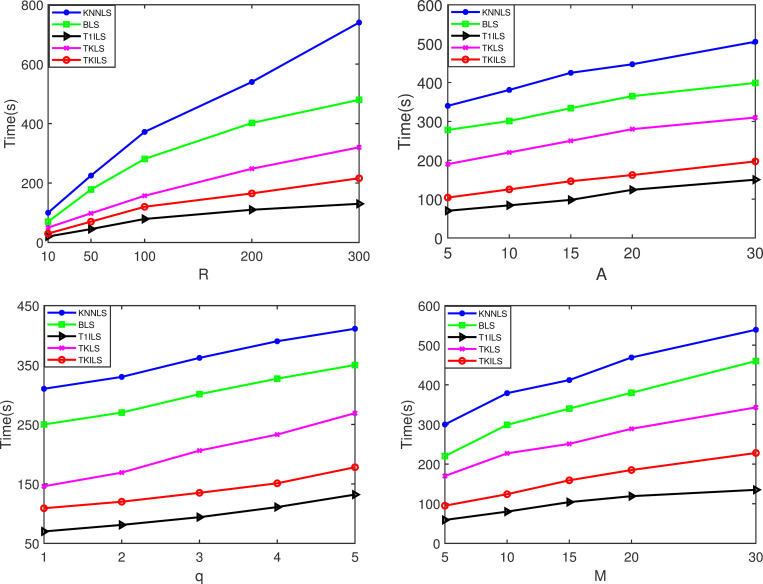
Time results on the synthetic datasets. Note: Fig 7(A)–7(D) are the time results for varying |*R*|, |*A*|, |*q*| and |*M*| on the synthetic datasets, respectively.

**Fig 8:**
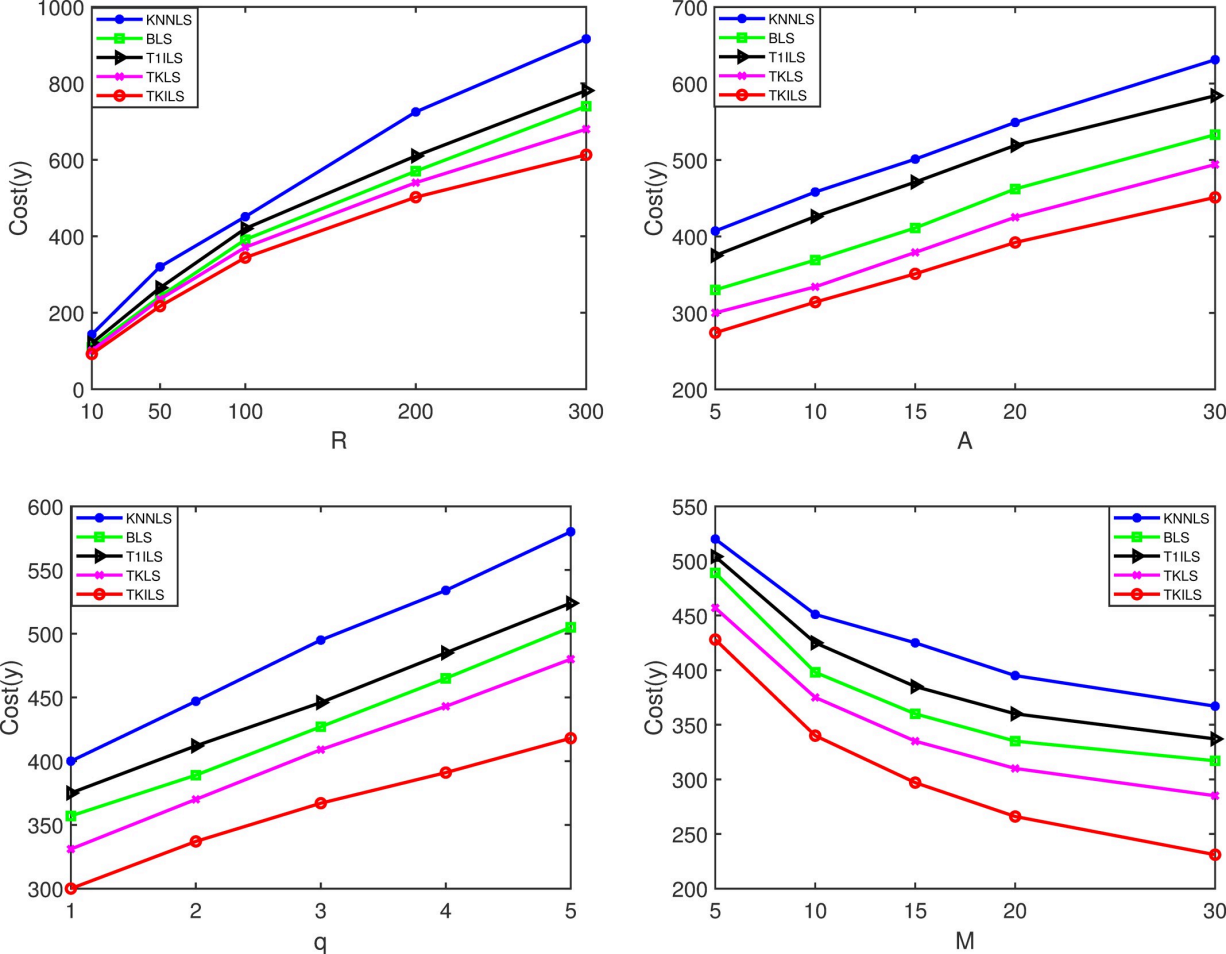
Cost results on the synthetic datasets. Note: Fig 8(A)–8(D) are the cost results for varying |*R*|, |*A*|, |*q*| and |*M*|, on the synthetic datasets, respectively.

Figs 7 and 8 show the experimental results of the five algorithms on the synthetic dataset. As shown in Fig 7(A)–7(D), TKILS and T1ILS with improved local search have good running performance. The reason is that the improved local search uses the greedy cost function to generate the initial distribution path, which has a great improvement in the quality of the initial solution compared with the random generation. This enhancement reduces the local search optimization process for the initial solution, further reducing the running time of the algorithm. Similarly, TKILS and TKLS algorithms consisting of Top-K recommendation perform significantly better than KNNLS and BLS because, compared with K-Nearest Neighbor search and Breadth-first search, Top-K recommendation reduces the search volume of a large number of unsatisfied solutions under the restriction of search level *ε*. T1ILS has the fastest running time among all algorithms because the Top-1 recommendation used largely reduces the selection space of solutions compared to the Top-k recommendation.

Fig 8(A)-8(D) show that TKILS has the optimal order delivery cost in terms of solution execution cost. This is because in case the order is not fully fulfilled, KNNLS needs to keep searching for the closest store to the order, which can easily lead to unlimited order-split, further leading to a significant increase in order fulfillment costs. Similarly, BLS needs to keep searching for stores that can maximize the order fulfillment, due to the lack of consideration of the delivery distance between stores and orders, which tends to miss the lower cost delivery plan. In contrast, the TKILS proposed in this paper combines the advantages of KNNLS and BLS. The order fulfillment plans are first generated based on the minimum number of splits, and then the schemes are ranked according to the delivery distance. In other words, TKILS can control the number of order-split as well as ensure a reasonable delivery cost. Likewise, it can be seen by Fig 8(D) that the cost reduction of TKILS is significant as the number of stores increases. This is because the improved local search utilizes 0-exchange, 0-reset for collaborative store-to-store delivery. The overlapping delivery paths are effectively reduced by order scheduling.

#### 3.2.2 Results on the real datasets

**Fig 9:**
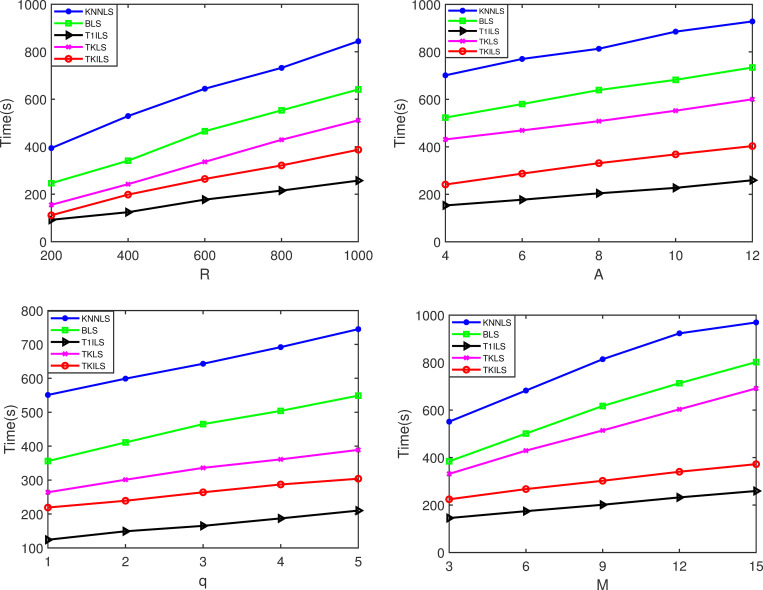
Time results on the real datasets. Note: Fig 9(A)–9(D) are the time results for varying |*R*|, |*A*|, |*q*| and |*M*| on the real datasets, respectively.

**Fig 10:**
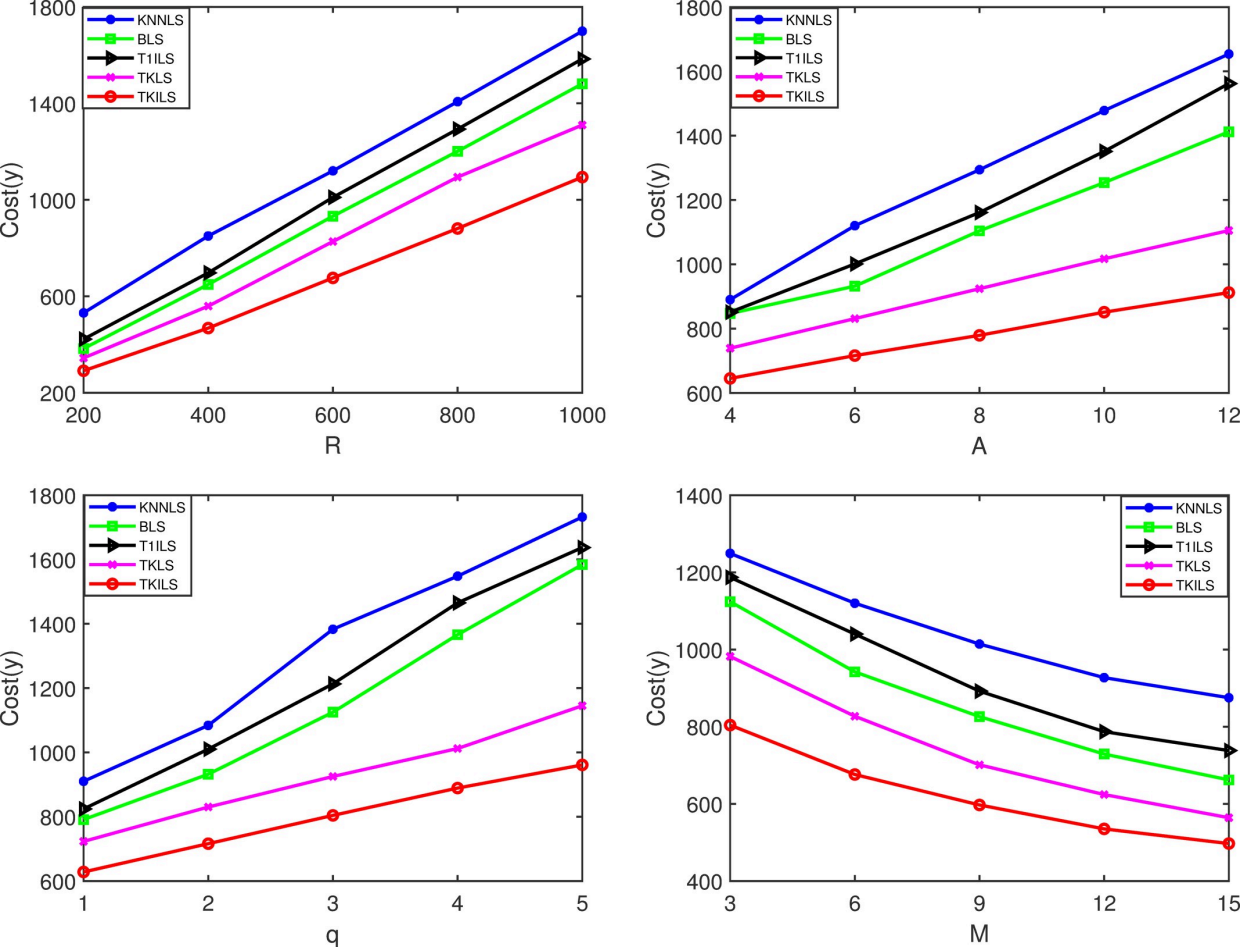
Cost results on the real datasets. Note: Fig 10(A)–10(D) are the cost results for varying |*R*|, |*A*|, |*q*| and |*M*|, on the real datasets, respectively.

Figs 9 and 10 are the experimental results on the real datasets. As the order size increases, the five algorithms perform similarly to the results on the synthetic dataset, which indicates that the structure of each algorithm is stable in the range of 1000 orders. For TKILS, within 1000 orders, the optimal solution in POOL will remain at 3000, at which order size the improved local search can run quickly. The computation time and execution cost of all algorithms, in the real context of 600 fixed orders, are higher than the results on the synthetic dataset, indicating that the order size has a significant impact on the performance of the algorithms. Among them, TKILS has the best combined results. This is because TKILS has the following characteristics: the smaller the number of solutions in POOL the faster the computation speed, and the larger the number of solutions the lower the execution cost. That is, by increasing a certain amount of computing time to achieve a significant reduction in cost, which is relevant for companies that need to reduce the order fulfillment cost.

#### 3.2.3 Feasibility of the algorithms

**Fig 11:**
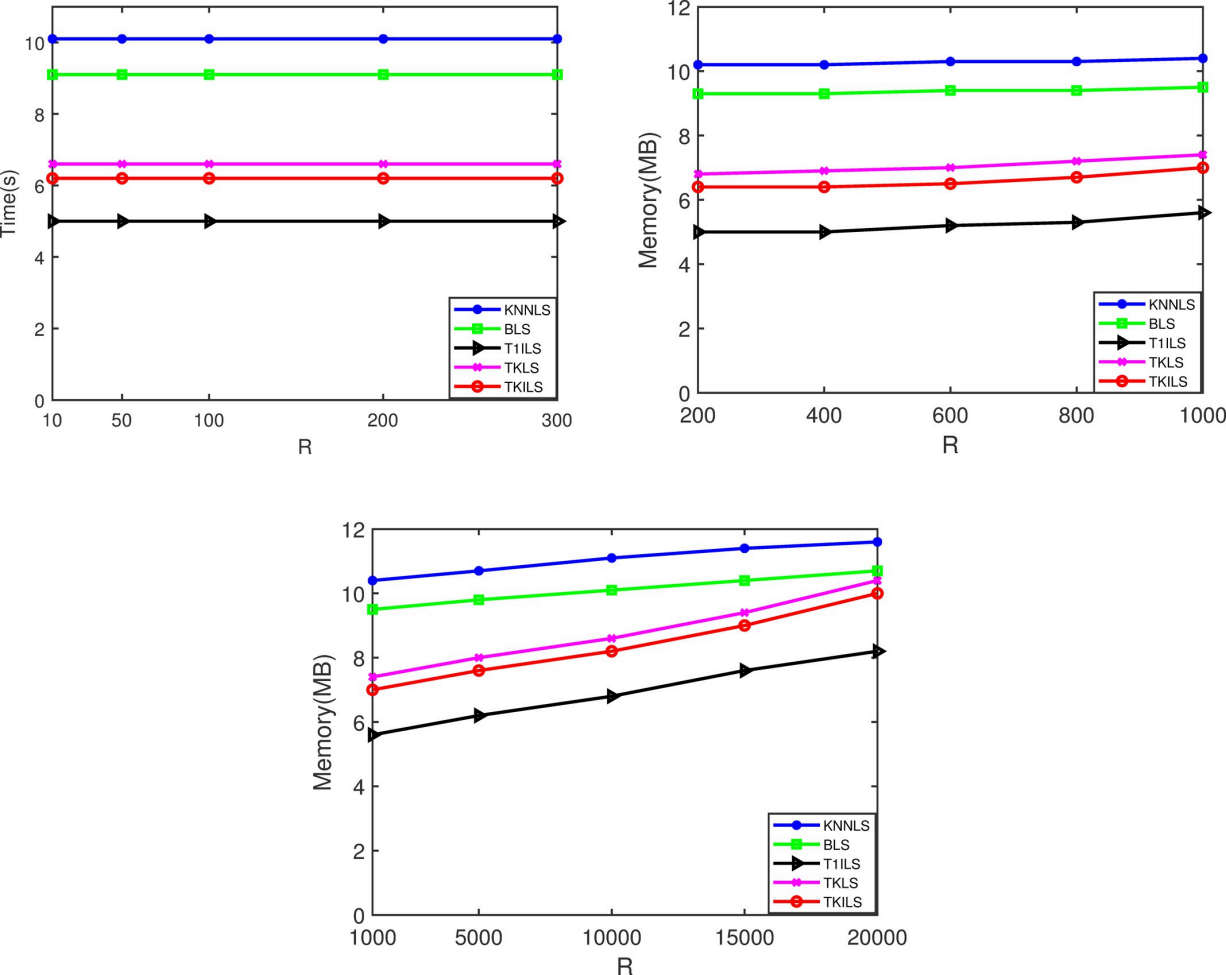
Running memory of the algorithms. Note: Fig 11(A)–11(C) are the running memory results for varying |*R*| from 10 to 20000 on the real datasets, respectively.

To ensure the smooth operation of the algorithm, this study calculated the running memory occupied by the algorithm at three order sizes [10, 200] [200, 1000] [1000, 20000], the results are shown in Fig 11. From Fig 11(A) and 11(B), it can be seen that the memory occupied by each algorithm is almost constant when the order sizes are [10, 200] and [200, 1000]. This is because the number of order plans for each algorithm at small order sizes is smaller and less difficult to compute. It is consistent with the performance of the algorithms in terms of running time and execution cost in the previous experiments. As the order size scales up to [1000, 20000], the memory of all algorithms starts to change visibly, and the memory increase for TKILS and TKLS is especially significant after the order size reaches more than 10000. The reason is that Top-K recommendation needs to store 3 plans for each order. The larger the order size, the more alternatives there are in POOL, resulting in a more difficult computation and a larger memory footprint for the run. This indicates that TKILS is more suitable for order sizes less than 10000.

### 3.3 Summary of results

According to 3.2 Experimental results, T1ILS has the best performance in terms of the running time, and TKILS has the best performance in terms of the execution cost. KNNLS and BLS are far less effective than TKLS, T1ILS and TKILS in handling small-scale orders because they only perform simple searches. The running time of TKILS is only after T1ILS, and the running memory occupied by TKILS during operation is always within a reasonable range. Therefore, when the order size is small, the combined effect of TKILS is the best.

It is important to recognize that the performance of TKILS decreases as the order size further expands, due to the exponentially increasing number of split-plans caused by the order expansion. Specifically, TKILS takes a multi-alternative order-split strategy, where each order has K feasible optimal order-split solutions. This approach leads to too many alternatives stored in the POOL, thus occupying a larger amount of memory. As a result, it takes more time to obtain the optimal delivery path to achieve optimization of order fulfillment cost.

With the continuous development of new retailing, a large number of traditional retailers are expanding their online business, and many e-tailers are moving into offline physical retailing too. TKILS can help companies with physical stores to plan the distribution and delivery of orders within a certain geographical area. The TKILS can be embedded into the enterprise’s online retail platform. When a new order appears, use TKILS to split the order and get the order fulfillment stores. Through the joint optimization between order-split and order delivery, as well as the collaborative delivery between stores, to improve the delivery efficiency of each store, finally achieve the goal of minimizing order execution cost.

It is known that for retailers, the reduction of order fulfillment cost plays an important role in running the business and increasing profits. Since TKILS has the best order fulfillment cost for any order size, it can be concluded that TKILS is the most suitable algorithm.

## 4. Conclusion

New retailing leads to the development of the order fulfillment model with physical stores as front warehouses. In order to satisfy the online order processing needs of physical stores, this study presents a new problem, called "the Multi-Store Collaborative Delivery Optimization (MCDO)". To solve the problem, a hybrid heuristic algorithm is proposed, which combines width-first search and local search.

This study limits the upper limit of the width-first search by level restriction *ε*, which effectively reduces the search time, and improve the efficiency and effectiveness of splitting orders by introducing Top-K alternatives. Firstly, improve the initial solution of the local search using the greedy cost function to reduce the number of iterations of the algorithm. Then optimize the delivery routes through alternative order solutions to reduce the order fulfillment cost through collaborative delivery among stores. Extensive experiments on synthetic and real datasets show that our proposed algorithm outperforms applicability and effectiveness by a large margin.

After study it is found that in experiments TKILS has the best execution cost, and the running time and running memory are also in a reasonable range. However, TKILS suffers from a sharp increase in runtime and runtime memory as the order size continues to grow. Moreover, since the number of stores is fixed, the advantage of TKILS to reduce order fulfillment cost by increasing stores becomes smaller compared to other algorithms. However, for retailers who need to reduce order fulfillment costs, it is acceptable to spend more time to get order fulfillment results, which means that TKILS is a good tool in reducing order fulfillment costs.

## Supporting information

S1 File Data set. All experimental data are included.
(ZIP)
